# Reviewed article: Souza MA, Lopes LC, Silva MT. Factors associated with complications related to the use of peripherally inserted central venous catheter in hospitalized children: a case-control study, Sorocaba, Brazil, 2018-2022. Epidemiol Serv Saude. 2025:34;e20240134

**DOI:** 10.1590/S2237-96222025v34e20240134.a

**Published:** 2025-09-08

**Authors:** Catiane Costa Viana

**Affiliations:** 1 Universidade Federal de Minas Gerais

**Table:** 

Rating	Excellent	Good	Average	Below average	Poor
Interest	✓				
Quality			✓		
Originality		✓			
Overall		✓			

**Table:** 

Questionnaire	Yes	No	Not applicable
Does the manuscript contain new and significant information to justify publication?	✓		
Does the Abstract (Summary) clearly and accurately describe the content of the article?		✓	
Is the problem significant and concisely stated?	✓		
Are the methods described comprehensively?		✓	
Are the interpretations and conclusions justified by the results?		✓	
Is adequate reference made to other work in the field?		✓	

## Manuscript Structure

Length of article is: Adequate

Number of tables is: Adequate

Number of figures is: Adequate

Please state any conflict(s) of interest that you have in relation to the review of this paper (state “none” if this is not applicable): None

## Comments

Comentários ao Autor

Este estudo caso-controle aborda uma importante área de pesquisa. As complicações relacionadas ao uso de cateter venoso central de inserção periférica (PICC) podem prolongar o tempo de internação hospitalar, aumentar a mortalidade e os custos da internação. A compreensão dos fatores associados à ocorrência dessas complicações pode contribuir para o desenvolvimento de ações voltadas à redução dessas complicações. Essas ações podem beneficiar os pacientes, as instituições e o Sistema Único de Saúde. No entanto, há pontos no estudo que precisam de atenção, como mencionados abaixo.

Comentários críticos ao autor

- Nas considerações éticas, forneça detalhes sobre o consentimento dos participantes. Informe se o consentimento dos pais ou responsáveis dos participantes foi obtido antes da realização do estudo.
- A lacuna do conhecimento a ser preenchida (página 8, linhas 11-18) está relacionada à “identificação dos fatores associados as complicações relacionadas ao uso do PICC em crianças submetidas a procedimento cirúrgicos”. No entanto, o objetivo apresentado no final da introdução é “verificar os fatores associados a ocorrência de complicações durante o uso do PICC, considerando a população pediátrica hospitalar”. Sugiro ajustes nessas informações, para manter a concordância entre a justificativa para realização do estudo e o objetivo proposto. Deixar claro no objetivo se as complicações de interesse são relacionadas ao uso do PICC e se a população de interesse são crianças submetidas a cirurgias. Essas informações também precisam estar claras nos métodos e nos resultados apresentados. Esclareça as hipóteses pré-especificadas.
- Revise as conclusões (no resumo e no final do artigo) para garantir que sejam justificadas pelos resultados e respondam diretamente à pergunta de pesquisa. Evite conclusões que extrapolam os resultados. A conclusão também deve vincular-se ao escopo da revista: o uso da epidemiologia para o aprimoramento das ações de vigilância e dos serviços de saúde.
  Comentários importantes ao autor
- Sugiro ajuste no título para que fique de acordo com o objetivo e reflita o conteúdo do manuscrito, de forma mais clara e o mais objetiva possível.
- Sugiro que o resumo seja revisado para manter a concordância, de maneira precisa e concisa, com o trabalho realizado. O objetivo no resumo está diferente do objetivo apresentado no final da introdução. As descrições dos casos e controles também não estão de acordo com o que foi apresentado no trabalho. Sugiro incluir valores odds ratio e respectivos intervalos de confiança para os resultados apresentados.
- Descreva os métodos com mais detalhes, para que outros pesquisadores possam replicar o estudo. A descrição dos métodos precisa estar concordante com o guia de redação de estudos caso-controle (ver instruções aos autores).
- Página 7, linha 48: a informação ”as complicações mais comuns decorrentes da remoção do PICC” não seria “as complicações mais comuns decorrentes do uso do PICC”?
- Página 10, linhas 4 a 7: Esclarecer a informação “com análise dos pacientes que apresentaram complicações inerentes à inserção do cateter”.
- Página 12, linha 11: foi citada a Resolução 196/96 do Conselhos Nacional de Saúde. Essa Resolução foi revogada em 2012. Sugiro alteração para a Resolução 466, de 12 de dezembro de 2012, que é a vigente.
- Nos métodos, sugiro listar todas as complicações relacionadas ao uso do PICC, que são de interesse no estudo. Indique quais critérios ou métodos foram utilizados para a determinação dessas complicações.
- Nos métodos estatísticos esclareça qual o resultado de interesse do estudo.
- Foi realizado cálculo amostral? Esclareça qual critério utilizado para selecionar e definir o tamanho do grupo controle.
- Os resultados no resumo e no artigo precisam responder ao objetivo do estudo. O que foi proposto no objetivo (questão central do estudo) foi descrito nos resultados como “achados secundários” e como “outros achados”. Entendo que esse deveria ser o resultado principal do estudo (ver guia de redação de estudos caso-controle, que consta nas instruções aos autores, e últimos artigos publicados na revista como exemplos).
- Na figura 1, verifique a concordância dos critérios de inclusão e exclusão com o que foi apresentado nos métodos. A última categoria de idade considerada nos métodos foi “até 2 anos”, enquanto na Figura 1, o critério de exclusão foi “Crianças maiores de 3 anos (n=13)”. Sugiro rever se a categoria “Até 2 anos” não seria de 1 ano e um dia até 2 anos.
- Na Figura 1, o critério de exclusão “Passagem de CVC (n= 194)” não foi mencionado nos métodos. Além disso, os pacientes controles foram divididos em alta hospitalar (n= 28), desvio de qualidade (n= 9) e contrarreferência com PICC (n=10). Esses critérios não foram descritos nos métodos.
- Sugiro revisão das porcentagens apresentadas na tabela 1. O valor N considerado para o cálculo das porcentagens nas colunas dos casos e dos controles foi igual a 109. Não deveria ser considerado o valor N correspondente a cada coluna (62 e 47)? Isso permitiria melhor visualização das características dentro de cada grupo. Verificar também se para o cálculo das porcentagens nas tabelas 2 e 4 não deveriam ser considerados os valores N utilizados nas respectivas análises.

Comentários desejáveis ao autor

- Na introdução, sugiro verificação em literatura nacional e internacional sobre dados epidemiológicos atuais disponíveis sobre a magnitude do problema estudado. Informe o ano ou período de todos os dados apresentados, evitando expressões pouco específicas, como “atualmente”, “nas últimas décadas” e “nos últimos anos”. Qual o impacto das complicações relacionadas ao uso do PICC, para os pacientes e para os sistemas de saúde?
- Página 9, linhas 20 a 23: Sugiro o acréscimo de uma breve descrição da recertificação de Acreditação com Excelência, nível 3, da Organização Nacional de Acreditação, citando a referência consultada.
- Página 12, linhas 28-39: Sugiro mover as informações sobre as análises realizadas para a seção dos métodos.
- A estrutura do artigo pode ser melhorada (ver últimos artigos publicados na revista como exemplos). Em relação à discussão, sugiro apresentar um breve resumo dos principais achados, enfatizando como eles podem contribuir para ampliar o conhecimento sobre o assunto. Discuta as possíveis explicações sobre os resultados, comparando-os com resultados de outros trabalhos publicados. Discuta as limitações do estudo e dos métodos usados para minimizar as limitações. Na conclusão, exponha as implicações dos resultados do estudo para os serviços de saúde e/ou para as políticas de saúde.
- Revise a lista de referências citadas, verificando se foram incluídos trabalhos mais relevantes e recentes (até 5 anos) sobre o tema.
- O texto precisa ser revisado para garantir clareza, precisão e objetividade.
- O texto precisa ser revisado para correção de erros gramaticais e ortográficos.

